# Qualitative analysis of a family satisfaction in an adult ICU

**DOI:** 10.1186/cc13218

**Published:** 2014-03-17

**Authors:** C Ebm, D Dawson, M Cecconi, A Rhodes

**Affiliations:** 1 Wiener Privatklinik, Vienna, Austria; 2 St George's NHS Trust, London, UK

## Introduction

Patient satisfaction is a key determinant of the quality of care within the hospital environment. Critically ill patients often lack capacity. Under such circumstances, family members are often main decision-makers and their satisfaction about the ICU experience resembles an important surrogate in evaluating the quality of care. We aimed to measure family satisfaction with different aspects of medical care.

## Methods

In February 2013, surveys, including 24 qualitative questions, were sent out to relatives of 50 patients 1 month after their discharge from ICU St George's, UK. Responses were graded 1 to 5, with higher values representing a greater degree of satisfaction.

## Results

**Figure 1:**
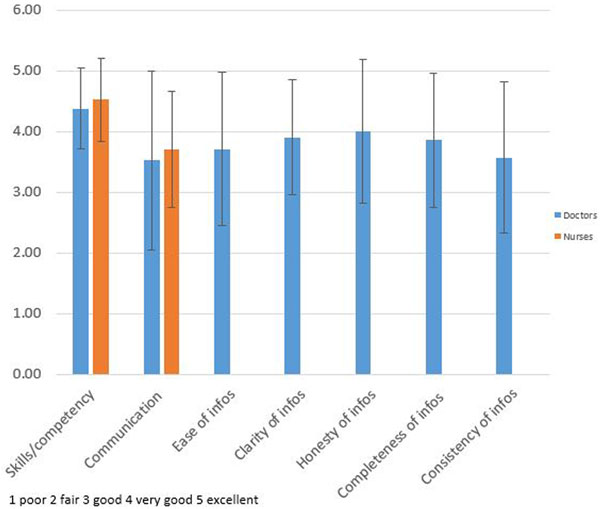
**Team performance**.

Most respondents were satisfied with the overall performance and the respect received (mean ± SD score 4.7 ± 0.68). Skills and competency of doctors (4.38 ± 0.67) and nurses (4.57 ± 0.68) was also perceived very positive. However, family satisfaction with communication with doctors (3.52 ± 1.47) and nurses (3.71 ± 0.96), as well as inclusion in decision-making (3.39 ± 1.24), resulted in somewhat lower scores. In particular, ease, clarity, consistency, honesty, and completeness of information given by doctors resulted in inhomogeneous perceptions between relatives (Figure 1). Additionally, the majority of responders felt only moderately satisfied with time and support to make a decision.

## Conclusion

In general, relatives felt very satisfied with the ICU, especially with the care of the patients and the professional workforce. The complete decision-making process was rated moderately good, which highlights some areas of improvement in involving relatives in the care of their beloved by providing regular, clear, easy to understand and consistent information.

